# Targeting DNA Methylation in Leukemia, Myelodysplastic Syndrome, and Lymphoma: A Potential Diagnostic, Prognostic, and Therapeutic Tool

**DOI:** 10.3390/ijms24010633

**Published:** 2022-12-30

**Authors:** Lenka Kalinkova, Aneta Sevcikova, Viola Stevurkova, Ivana Fridrichova, Sona Ciernikova

**Affiliations:** Department of Genetics, Cancer Research Institute, Biomedical Research Center of Slovak Academy of Sciences, 845 05 Bratislava, Slovakia

**Keywords:** hematologic malignancies, epigenetic regulation, DNA methylation, epi-drugs, hypomethylating agents, cancer treatment

## Abstract

DNA methylation represents a crucial mechanism of epigenetic regulation in hematologic malignancies. The methylation process is controlled by specific DNA methyl transferases and other regulators, which are often affected by genetic alterations. Global hypomethylation and hypermethylation of tumor suppressor genes are associated with hematologic cancer development and progression. Several epi-drugs have been successfully implicated in the treatment of hematologic malignancies, including the hypomethylating agents (HMAs) decitabine and azacytidine. However, combinations with other treatment modalities and the discovery of new molecules are still the subject of research to increase sensitivity to anti-cancer therapies and improve patient outcomes. In this review, we summarized the main functions of DNA methylation regulators and genetic events leading to changes in methylation landscapes. We provide current knowledge about target genes with aberrant methylation levels in leukemias, myelodysplastic syndromes, and malignant lymphomas. Moreover, we provide an overview of the clinical trials, focused mainly on the combined therapy of HMAs with other treatments and its impact on adverse events, treatment efficacy, and survival rates among hematologic cancer patients. In the era of precision medicine, a transition from genes to their regulation opens up the possibility of an epigenetic-based approach as a diagnostic, prognostic, and therapeutic tool.

## 1. Introduction

Hematologic malignancies form a heterogeneous group of acute and chronic diseases, clonally expanding into the blood, bone marrow, and lymph nodes. Due to their highly aggressive manner, blood cancers are characterized by rapid progress, affecting both pediatric and adult patients. Improved treatment response and outcomes have been documented in pediatric patients, who achieve remission in the overwhelming majority. Genetic and epigenetic changes lead to the clonal proliferation of stem and progenitor cells. Alterations in downstream signaling pathways contribute to the disruption of the self-renewal ability of hematopoietic cells and their differentiation into other lineages.

Although epigenetic mechanisms have been largely evaluated in embryonic development, differentiation, and organogenesis [1,2], mounting evidence supports their critical role in cancer development and treatment. In a very recent concept, aberrant epigenetic programming is one of the hallmarks of malignant progression [3]. The process of DNA methylation is the most studied epigenetic mechanism in both normal and cancer cells, and its correct regulation is crucial for the transcription of different regulating genes, maintaining genome integrity, and proper immune responses [4]. Genetic changes in DNA methylation enzymes and regulators together with abnormal DNA methylation are associated with cancer-promoting changes [5]. In addition to gene silencing by promoter hypermethylation at CpG islands, DNA genome hypomethylation is a typical sign of human cancers [4].

DNA methylation plays a critical role in hematopoiesis and hematopoietic stem cell differentiation and proliferation [6]. Epigenetic modulation by DNA methylation is involved in several stages, including blood cell lineaging and the formation of the final cell types [7]. According to the findings, aberrant DNA methylation patterns during hematopoiesis linked to the dysfunction of DNA methylation-related enzymes often lead to blood cancer development [8]. With heterogeneity and different clinical severity of individual subtypes, it is more than inevitable that investigations into new diagnostic and therapeutic possibilities are performed.

In this review, we provide current data focusing on the emerging role of DNA methylation-related changes in hematologic malignancies, including leukemias, myelodysplastic syndromes, and lymphomas. The studies concerning the aberrant expression of DNA methylation enzymes and target genes are analyzed. Moreover, we will discuss the therapeutic implication of DNA methylation status and provide an overview of the clinical trials, evaluating the clinical significance of DNA methylation mechanisms in hematological malignancies.

## 2. Enzymes and Regulators of DNA Methylation in Hematologic Malignancies

The process of DNA methylation is catalyzed by enzymes called DNA methyltransferases (DNMTs) that transfer a methyl group from S-adenosine methionine (SAM) to the fifth carbon of a cytosine. Five *DNMT* genes are known in the human genome, including *DNMT1*, *DNMT2*, *DNMT3A*, *DNMT3B*, and *DNMT3L*, but only de novo *DNMT3A*, *DNMT3B*, and *DNMT1* have a catalytic domain with methylation activity. There was much evidence proving that the regulation of DNA methylation by DNMTs is critical for normal hematopoiesis [9]. Changes in the methylation level can be the result of mutations causing gain or loss of function of the various enzymes involved in the methylation, including DNMTs, and other methylation modifiers such as ten-eleven translocation methylcytosine dioxygenases (TETs), isocitrate dehydrogenase 1/2; (IDH1/2), or others [10].

### 2.1. DNA Methyltransferases

During normal hematopoiesis, DNMT1 has an essential role in hematopoietic stem cell (HSC) self-renewal and multilineage hematopoietic differentiation, because the lack of DNMT1 in HSCs leads to a reduction in myeloid lineages compared to lymphoid lineages [11]. Together with other DNMTs, DNMT1 was substantially overexpressed in leukemia cells specifically according to leukemia type; likewise, acute myeloid leukemia (AML) cells with methylated *p15(INAK4B)* expressed higher levels of DNMT1 and DNMT3B [12]. Overexpression of DNMT1 was observed in other hematologic malignancies, including T-cell acute lymphoblastic leukemia (ALL), T-cell lymphomas, and diffuse large B-cell lymphoma (DLBCL) [13,14,15]. Nevertheless, in humans, only a few mutations at very low frequencies in the *DNMT1* gene have been detected in AML cells [10].

Since DNMT1 represents one of the key methylation regulators, it could serve as a potential therapeutic target for lymphoid and myeloid malignancies. In chronic myeloid leukemia (CML), aberrant DNA hypermethylation contributes to low tyrosine kinase inhibitor (TKI) sensitivity and the persistence of leukemic stem cells (LSCs). Targeting DNMT1-mediated aberrant DNA hypermethylation using OR21 (orally available single-compound prodrug of decitabine) showed anti-tumor activity and impaired LSCs, suggesting it is a potential therapeutic target for CML [16]. DNMT3a and DNMT3b represent de novo DNMTs, which functionally cooperate in hematopoiesis and enable HSC differentiation. Although low-frequency mutations in the *DNMT3B* gene are found in human hematological malignancies, the role of DNMT3B was well documented in mouse hematopoiesis.

Lopusna et al. observed that 43% of Dnmt3b^+/−^ mice developed T-cell lymphomas, chronic lymphocytic leukemia (CLL), and myeloproliferative disorders. Dnmt3b^+/CI^ (CI, catalytically inactive) and Dnmt3b^CI/CI^ mice were affected by B-cell rather than T-cell malignancies. These observations could suggest that Dnmt3b is critical to prevent B-cell transformation in vivo [17]. Moreover, the overexpression of DNMT3B and its isoforms has been associated with poor prognosis in older AML patients [18]. However, contradictory studies were published regarding DNMT3b expression in hematological malignancies [19]. As documented, DNMT3a is essential for HSC differentiation. Challen et al. reported a decline in the differentiation capacity of DNMT3a-null HSCs, resulting in the accumulation of undifferentiated HSCs in the bone marrow [20].

In hematopoietic disorders and malignancies, a high prevalence of *DNMT3A* mutations has been reported, mainly in patients with AML [21,22]. Somatic mutations decrease DNMT3A activity, induce CpG hypomethylation, and may promote the transformation of hematopoietic to cancer cells [23,24]. The most frequent *DNMT3A* hot-spot mutation R882H was observed in up to 60% of AML [21,25], 18% of ALL [26], and 10% of myelodysplastic syndrome (MDS) patients [27]. This alteration is associated with an adverse prognosis since it reduces DNA binding at the homodimeric interface. Spencer et al. observed a focal methylation loss in non-leukemic hematopoietic cells with a *DNMT3A* mutation R882H, suggesting that this mechanism could precede AML. As noted, CpG island hypermethylation in patients with wild-type DNMT3A did not correlate with gene silencing. According to the authors, DNMT3A-mediated hypermethylation might be a consequence of AML progression [28]. Importantly, *DNMT3A* mutations in AML patients are associated with shorter overall survival (OS) and predict an improved outcome with high-dose induction chemotherapy, representing a promising prognostic and predictive tool [29,30].

### 2.2. Modifiers and Regulators of DNA Methylation

TET enzymes represent DNA demethylation regulators, which are involved in the removal of methyl marks from DNA and play a role in hematopoietic differentiation. *TET1* and *TET3* genetic changes are very rare in leukemia. However, *TET2* is one of the most mutated genes in AML, chronic myelomonocytic leukemia (CMML), and MDS [8]. In a cohort of 27 DLBCL patients, only 1 patient (3.7%) with a frameshift mutation in *TET2* was detected, suggesting a low mutation rate in DLBCL patients [31]. Patients with low *TET2* expression had markedly shorter OS in both non-M3 (hypergranular AML) and cytogenetically normal AML (CN-AML), with a significant correlation between *TET2* expression and OS in CN-AML patients [32]. Cimmino et al. documented a tumor-suppressor role of *TET1* and found alternative mechanisms of *TET1* inactivation in human B-cell non-Hodgkin lymphomas (NHL) [33]. In contrast, high *TET1* expression was independently associated with poor OS and complete remission (CR) in CN-AML patients [34].

IDH1/2 are enzymes that convert isocitrate to α-ketoglutarate, which could serve as a substrate for other enzymes, e.g., TET2. In a study on AML patient cohort, high-throughput resequencing revealed genetic alterations in *IDH1*, *IDH2*, and *TET2* with frequencies of 6.2%, 8.6%, and 7.3%, respectively [35]. As noted, all 45 differentially methylated regions (DMRs) were hypermethylated in AML patients harboring *IDH1/2* mutations. AML cells with mutations in *TET2* or *IDH1/2* displayed hypermethylation, suggesting its important function in hematopoietic cells among AML patients [35]. In contrast to AML, the incidence and prognostic value of *IDH1/2* mutations in T-cell acute lymphoblastic leukemia (T-ALL) are poorly reported. Simonin et al. provided the first analysis of the oncogenetic landscape of *IDH1/2* mutations in 1085 T-ALL patients. The results showed that almost 4% of *IDH1/2* mutations correlated with poor prognosis [36].

Additionally, other regulators of DNA hypermethylation, including CCAAT/enhancer binding protein α (CEBPA) and Wilms tumor protein 1 (*WT1*), were reported to increase methylation in AML patients. CEBPA physically interacts with DNMT3A, and the N321D mutation in *CEBPA* decreases the DNA-binding ability of DNMT3. Furthermore, *CEBPA* mutations are responsible for promoter hypermethylation of various genes, including Polycomb Repression Complex (PCR2) target genes active in histone H3 methylation [37].

## 3. DNA Methylation of Target Genes in Leukemias, Myelodysplastic Syndromes, and Lymphomas

Genetic changes in regulators of the DNA methylation process can cause alterations in genome-methylation levels in the form of a loss of global methylation. On the other hand, almost intact global methylation was found in several hematologic malignancies. Genome-wide methylation studies in AML, CML, CLL, ALL, and lymphomas described different regions with aberrantly methylated genes, which could help to elucidate the malignant transformation process and find potential targets for diagnosis, prognosis, or therapy of hematological cancers [38,39,40,41,42,43].

### 3.1. Aberrant Methylation in MDS and AML

**Figure 1:**
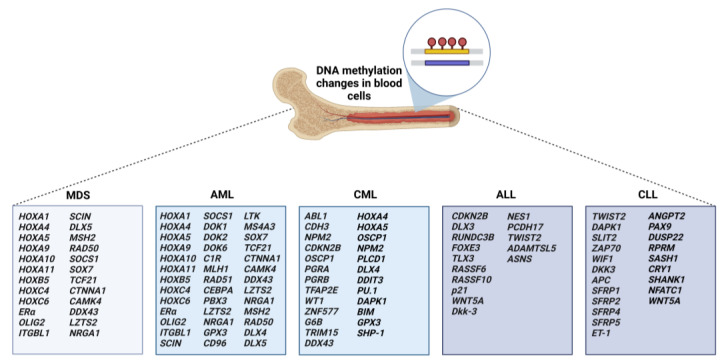
Methylation-affected genes associated with leukemias and myelodysplastic syndromes. DNA methylation plays a key role in the initiation and progression of hematological malignancies. DNMTs catalyze transferring of the methyl group (a red circle) to the 5-carbon position of cytosine within CpG dinucleotides in DNA sequence (each strand of double-stranded DNA is marked by different colors, yellow and blue), leading to the formation of 5-methyl-cytosine. Abnormal methylation patterns in bone marrow cells may predict responsiveness to the treatment. A panel of aberrantly methylated genes shown to have diagnostic and prognostic value. Targeting the hypermethylated promoters of tumor suppressor genes might represent a perspective trend for hypomethylating drug therapy alone or in combination. Abbreviation: DNMTs, DNA methyltransferases.

MDS and AML belong to the group of hematologic malignancies characterized by clonal hematopoiesis. Both diagnoses share similar clinical and pathologic features, but they differ in the percentage of blasts in peripheral blood and bone marrow. DNA methylation of different target genes has been described in patients with MDS and acute/chronic leukemia (Figure 1).

The *HOX* gene family represents the most studied genes, which are regulated by DNA methylation in AML. In an extensive study, Gao et al. identified 29 genes whose expression correlated with differently methylated CpG sites. Within the HOX family, high methylation was found in *HOXA7*, *HOXA9*, *HOXA10*, and *HOXB3* genes [38]. Similar results were found in mesenchymal stromal cells from bone marrow in MDS and AML patients, where preferentially aberrant methylated genes were *HOXA1*, *HOXA4*, *HOXA5*, *HOXA9*, *HOXA10*, *HOXA11*, *HOXB5*, *HOXC4*, and *HOXC6* [44].

Promoters of many tumor suppressor genes (TSGs) are most commonly inhibited by methylation. Cyclin-dependent kinase inhibitors *p15* and *p16*, important in the regulation of proliferation, are frequently methylated in AML patients. Their higher methylation was associated with lower OS, recurrence-free survival (RFS), and frequency of CR [45,46,47]. In AML and MDS patients, the relationship between gene methylation status and shorter OS was reported for *ERα*, *OLIG2*, *ITGBL1*, *SCIN*, *DLX5*, *MSH2*, *RAD50*, and *SOCS1* genes [48,49,50,51,52,53].

The DOK protein family, characterized as phosphotyrosine adapters with several functions in cell biology, is commonly expressed in myeloid cells. In a study comprising AML patients, He et al. observed that hypermethylation-mediated decreased expression of *DOK1* and *DOK2* genes was associated with lower OS [54]. Similarly, the higher methylation status of another member *DOK6* gene was found in AML patients, but with the opposite effect on OS [55]. Longer OS was associated with higher promoter methylation in other genes, including *C1R* and DNA repair genes *MLH1* and *RAD51* [52,56].

Sestakova et al. performed an extensive validation study for 27 genes from 14 studies to verify the predictive role of aberrant DNA methylation in AML. The results showed that hypermethylation of *CEBPA*, *PBX3*, *LZTS2*, and *NRGA1* serves as a predictor for longer survival [57]. In addition, higher methylation of *GPX3* and *DLX4* correlated with a favorable treatment impact, which is in contrast to previously reported studies documenting their correlation with lower OS [58,59].

In some cases, mutations in important transcription factors can lead to alterations in epigenetic modifications. Genetic changes in Runt-related transcription factor 1 (RUNX1), a key player in hematopoiesis, led to aberrant methylation in target genes. The study on the *RUNX1*-mutated AML cohort identified 51 differential methylated genes. As shown, the changes in expression profiles were found in ten of them. Hypermethylation of *CD96*, *LTK*, and *MS4A3* correlated with poor prognosis, which could be related to their effects on cell cycle regulation and differentiation [60]. Higher methylation of *SOX7*, *TCF21*, *CTNNA1*, and *CAMK4,* as well as hypomethylation of *DDX43*, *LZTS2*, or *NRGA1,* was observed in MDS/AML patients. Due to the lack of prognostic potential in numerous studies, complex evaluation requires further investigation [61,62,63,64,65,66].

### 3.2. Aberrant Methylation in CML

CML is characterized by the presence of translocation-creating oncoprotein BCR-ABL1, and the development of this disease consists of three phases, namely, the chronic phase (CP), accelerated phase (AP), and blast crisis phase (BP) [67]. The high content of aberrantly methylated CpG compared to healthy donors was found through a methylome analysis of CML patients. Moreover, the number of abnormally methylated CpG increased from CP to the BP phase. Most of the CpG sites with increased methylation (88%) were located in GpG islands or in the very close region, which overlapped with 348 genes in the peripheral blood of BP patients [39].

Jelinek et al. observed that among 10 selected genes analyzed by pyrosequencing, *ABL1*, *CDH3*, and *NPM2* presented the highest methylation in all phases of CML. Furthermore, increased methylation of *CDKN2B* (*p15*), *OSCP1*, *PGRA*, *PGRB*, and *TFAP2E* genes was described during CML progression [68]. Another extensive study defined 33 highly methylation-affected regions with hypermethylation of *ABL1*, *WT1*, *ZNF577*, and hypomethylation of *G6B* and *TRIM15* [69]. *DDX43* represents a frequently overexpressed gene in hematological malignancies. Epigenetic regulation of *DDX43* by promoter methylation and a negative correlation between hypomethylation and higher expression of *DDX43* was observed in CML patients. Moreover, the frequency of *DDX43* hypomethylation increased in CP, AP, and BP by 23.4%, 25.0%, and 33.3%, respectively [70].

Promoter hypermethylation is mostly associated with TSGs involved in crucial cell functions, including the regulation of differentiation, proliferation, apoptosis, cell cycle, and growth. Similar to AML, *HOXA4* and *HOXA5* were hypermethylated in CML patients, and the presence of promoter methylation correlated with resistance to imatinib, belonging to a group of TKIs. Patients with *HOXA4* and *HOXA5* methylation levels higher than 63% showed 3.78- and 3.95-times-higher risk for imatinib resistance, respectively [71]. Imatinib resistance was also recorded in the case of higher methylation of *OSCP1* and *NPM2* genes [68]. Aberrant methylation of TSGs, including *PLCD1*, *DLX4*, *DDIT3*, *PU.1*, *DAPK1*, *BIM*, and *GPX3,* could represent potential prognostic or therapeutic targets in CML [72,73,74,75,76,77,78]. Accordingly, the downregulation of *SHP-1* and relevant protein expression was associated with the presence of promoter methylation in advanced CML patients. SHP-1, a protein tyrosine phosphatase, is expressed mainly in HSC and plays a critical role in the regulation of JAK/STAT and MYC, AKT, and MAPK pathways. Alterations in *SHP-1* methylation status could lead to the deregulation of included pathways and blastic transformation in CML patients [79].

### 3.3. Aberrant Methylation in ALL

ALL represents hematological cancer of immature T or B cells. T-ALL and B-ALL represent approximately 15–20% and 85% of all cases, respectively [80]. A genome-wide methylation analysis, based on nine genes with the identified CpG methylator phenotype, was capable of predicting a poor outcome subgroup of adult T-ALL. Patients with low methylation levels reported shorter OS and a higher risk of death in univariate and multivariate analyses. According to the results, the lowest methylation levels in patients were significantly associated with gender, younger age, and a higher count of white blood cells [81]. Similar to AML, *CDKN2B* (*p15*) presented decreased expression in most T-ALL cases, originating from deletion and promoter hypermethylation. *CDKN2B* hypermethylation frequently occurred together with mutations in *DNMT3A* and *NRAS* genes. In addition to the association with an older age of onset, the results showed a relatively early presence of T-cell precursors of ALL, causing the quick arrest of T-cell differentiation [82]. *DLX3*, belonging to the *DLX* gene family with a wide range of functions during hematopoiesis, could be active in the resistance to apoptosis. In pediatric B-cell ALL, Campo Dell Orto et al. observed aberrant methylation of *DLX3* with reduced gene expression in patients with MLL-AF4 fusion, while no methylation was found in the subgroup with the TEL-AML1 fusion protein. The results suggested a potential role of *DLX3* methylation in B-cell acute leukemias [83]. Some epigenetic promoter alterations can be lineage-specific. Higher methylation and methylation-mediated downregulation of *RUNDC3B* expression are typical for lymphoid but not myeloid malignancies [84]. RUNDC3B participates in the MAPK cascade in the role of Rap2-MAPK signaling mediator. Silencing by promoter methylation could disrupt the MAPK signaling pathway and promote leukemogenesis of lymphoid cells [84].

Several specific methylation profiles were found to be diagnostic, prognostic, or therapeutic markers for ALL. Chatterton et al. monitored the methylation of *FOXE3* and *TLX3* genes, showing their ability to discriminate between cancerous and healthy bone marrow samples with high specificity and sensitivity, which indicates their potential as diagnostic markers. Furthermore, *TLX3* methylation correlated with minimal residual disease (MRD) in pediatric ALL patients [85]. In B-ALL and T-ALL-, *RASSF6* and *RASSF10* genes were frequently methylated and associated with the MRD in peripheral blood samples of adult ALL patients. In addition, the hypermethylation of *RASSF6* is significantly associated with shorter OS in precursor B-ALL patients [86,87]. In a series of further studies, Roman-Gomez et al. described the role of promoter hypermethylation in a prognostic manner while observing the association of higher methylation of *p21*, *WNT5A*, *Dkk-3*, and *NES1* genes with shorter disease-free survival (DFS) and OS [88,89,90,91]. Higher methylation of the *PCDH17* gene was frequently observed in both B-ALL and T-ALL with a relationship with lower OS and increased risk for relapse and death [92,93].

Importantly, the specific gene methylation profiles can represent a predictive therapeutic marker because aberrant methylation can be responsible for chemotherapeutic resistance. *TWIST2* hypermethylation and its inactivation were observed in more than 50% of ALL patients and 91% of samples from relapsed patients. In vitro experiments showed that the re-expression of TWIST2 increased apoptosis and sensitivity to chemotherapeutics [94]. A comparison of chemo-resistant and sensitive B-ALL pediatric patients detected higher levels of methylation in *ADAMTSL5* (93% vs. 38%) and *CDH11* (79% vs. 40%) in chemo-resistant vs. chemo-sensitive patients, respectively [95]. On the other hand, hypomethylation of the *ASNS* gene in T-ALL childhood patients was associated with poor outcomes and resistance to asparaginase, which is a high-dose drug involved in T-ALL therapy [96].

### 3.4. Aberrant Methylation in CLL

CLL belongs to the most common leukemias in the adult population, characterized by the clonal expansion of malignant B cells [97]. Similar to some other hematological malignancies, global hypomethylation is a characteristic sign of CLL. However, aberrantly methylated regions were characterized for this diagnosis in previous years [98]. In a genome-wide methylation study, Pei et al. identified approximately 1764 known genes with different methylation at 5’ regulatory regions. Among them, the results showed the presence of aberrant methylation in all four *HOX* gene clusters [40]. Previously, several studies found higher promoter methylation of genes, including *TWIST2*, *DAPK1*, *SLIT2*, or *ZAP70* [99,100,101,102]. Increased expression of *ZAP70* predicts poor outcomes for CLL patients, and CpG sites important for the regulation of transcription were identified in the 5’ regulatory region. According to these findings, a decreased methylation level in this specific CpG dinucleotide is a predictive biomarker for poor prognosis [102]. The WNT signaling pathway is generally involved in carcinogenesis and leukemogenesis. The constantly activated WNT pathway associated with the detection of hypermethylation of the seven WNT antagonist genes *WIF1*, *DKK3*, *APC*, *SFRP1*, *SFRP2*, *SFRP4*, and *SFRP5* was observed in the peripheral blood of CLL patients. However, no association between methylation and clinical parameters was confirmed [103,104].

The hormone peptide Endothelin-1 (ET-1) plays a role in various cell functions, including proliferation. Microenvironment stimuli activated downstream receptors, leading to increased *ET-1* expression in CLL. The unmethylated *ET-1* gene was observed in healthy donors, while CLL patient samples exhibited 32% unmethylated and 68% methylated profiles. As shown, high methylation of the first *ET-1* intron decreased its expression, suggesting the importance of epigenetic regulation [105]. The same authors detected that low methylation levels of neoangiogenic factor *ANGPT2* correlated with increased expression and are associated with shorter OS and poor prognosis in CLL patients [106]. The prognostic potential of aberrant methylation in CLL was also observed in other genes, including *PAX9*, *DUSP22*, *RPRM*, *SASH1*, and *CRY1* [107,108,109]. In the low-risk CLL subgroup, the hypermethylated *CRY1* promoter inactivated its expression and was associated with better outcomes [109]. For CLL diagnosis, the most differentially methylated gene *SHANK1* positively correlated with the absolute lymphocyte count. Increased *SHANK1* methylation was found in samples during the pre-CLL diagnosis period, suggesting that epigenetic modification of *SHANK1* occurred early in CLL carcinogenesis [110]. In comparison with *SHANK1*, the *NFATC1* gene belongs to the most hypomethylated genes identified in CLL. A decreased methylation level is strongly associated with *NFATC1* upregulation, resulting in the deregulation of target gene expression. Inactivation of NFAT regulator calcineurin by ibrutinib increased apoptosis in leukemic cells. Thus, *NFACT1* might represent a potential therapeutic target in CLL diagnosis [111].

Two subgroups of CLL patients are classified according to the presence of somatic mutations in immunoglobulin *(Ig)* genes. The Ig heavy chain variable region (IGHV) with high mutational prevalence (IGHV-M) correlated with a more favorable prognosis compared to IGHV unmutated (IGHV-UM) CLL patients [112]. In the IGHV-UM subgroup, *VHL* and *ABI3*, acting as TSGs, were preferentially methylated and correlated with decreasing expression [113]. In contrast, the expression of *WNT5A* distinguished patients with worse outcomes in the IGHV-M subgroup. Reduced *WNT5A* expression through hypermethylation preferentially in three CpG dinucleotides within the regulatory region correlated with good prognoses [114].

### 3.5. Aberrant Methylation in Malignant Lymphomas

Malignant lymphomas (MLs) represent a heterogeneous group of hematologic malignancies in primary or secondary lymphatic organs arising from various types of B and T lymphocytes or NK cells. Generally, MLs cover classical Hodgkin lymphomas (HL) and diverse groups of NHL [115]. Pathogenesis of HL is presumably associated with family anamnesis or infection with Epstein–Barr virus (EBV). According to the previous findings in HL, DNA methylation led to the silencing of *RASSF1A*, *p16INK4a*, *p18INK4c, p15INK4b*, *SYK*, *BOB*.*1/OBF*.*1*, and *CD79B* [116,117,118]. Recently, differences in DNA methylation signatures were detected in a study with monozygotic triplets with HL. Two of the triplets with HL shared DNA methylation changes in naive B-cells and marginal zone-like B-cells compared to a healthy non-HL-triplet. Hypermethylation of one region within chromosome 18 in naive B-cells was found exclusively in HL triplets [119].

Bethge et al. identified 233 downregulated genes in a cohort of B-cell NHL patients. From the analyzed gene panel, *DSP*, *FZD8*, *KCNH2*, and *PPP1R14A* exhibited promoter methylation in 28%, 67%, 22%, and 78%, respectively. In addition, the highest methylation level after treatment with demethylating agents was detected in *LRP12* and *CDH1* genes, presenting 94% and 92%, respectively [120,121]. Another study evaluated eight genes associated with lymphoma pathogenesis and found decreased *SIRT1* and increased *KLF4*, *DAPK1*, and *SPG20* gene methylation levels. In vitro analysis revealed that DNMT1 did not affect hypermethylation maintenance of *KLF4*, *DAPK1*, and *SPG20* genes [122,123].

**Figure 2:**
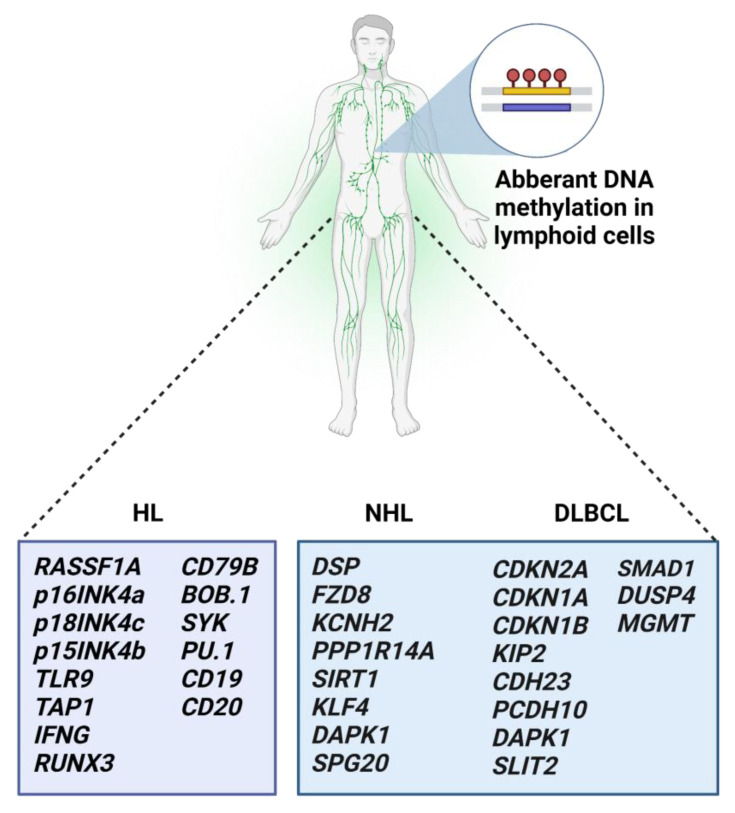
Methylation-affected genes associated with lymphomas. Aberrant DNA methylation (a red circle) regulates gene expression in Hodgkin, Non-Hodgkin, and Diffuse large B-cell lymphomas. Abnormally methylated genes might be used as potential biomarkers for therapeutic decisions and as predictive markers for patient outcomes.

Most DNA methylation studies have been performed in NHL, specifically in DLBCL (Figure 2). The results from the genome-wide methylation study reported approximately 200 differentially methylated genes in DLBCL patients with the aberrant methylation of *p16/CDKN2A*, *p21/CDKN1A*, and *p27/CDKN1B* [124]. However, only 37% of DLBCL patients had *p16* methylation higher than 5%. According to the results, patients younger than 65 years manifested better progression-free survival (PFS) when the *p16* methylation level reached more than 25% [125]. Aberrant methylation of another from CDK inhibitors, *p57*/*KIP2,* suggested that epigenetic modification of *p57* could be established as a biomarker for MRD in DLBCL [126]. Shawky et al. analyzed the panel of 20 TSGs for promoter hypermethylation and correlation with clinical characteristics and patient outcomes in the DLBCL group. The methylation of several studied genes associated with survival and chemoresistance, specifically *RUNX3*, *DAPK1*, and *MT16*, represent prognostic factors for DFS. Moreover, hypermethylation of *RUNX3* and *CDH1* was shown to be an independent prognostic factor for OS [127].

Promoter methylation analysis of genes coding cadherins and protocadherins uncovered the association of *CDH23* and *PCDH10* hypermethylation and downregulated expression with worse outcomes in DLBCL patients. As noted, methylation of these genes could serve as a risk marker or a potential therapeutic target [128,129]. Several methylation studies investigated the *DAPK1* gene showing higher methylation significantly associated with lower OS, disease-specific survival, and 5- year survival in the DLBCL patient cohort [130,131]. In addition, a prognostic and predictive potential of increased *DAPK1* methylation in plasma samples was revealed in DLBCL. Patients with decreased methylation levels survived longer than patients with unchanged or regained *DAPK1* methylation [132]. Specific promoter methylation with prognostic significance for DLBCL patients was discovered in several other genes, including *SLIT2*, *DUSP4*, and *MGMT* [133,134,135]. Importantly, Clozel et al. documented the link between aberrant DNA methylation and resistance to chemotherapeutics. Methylation analysis in chemoresistant DLBCL patients found nine hypermethylated genes. Among them, *SMAD1* was a critical player. In a clinical trial on DLBCL patients, treatment with azacitidine followed by chemoimmunotherapy showed demethylation of *SMAD1* and increased chemosensitivity [136].

## 4. Therapeutic Implication of DNA Methylation-Based Approach

Both global hypomethylation and specific promoter hypermethylation, primarily leading to the downregulation of TSGs, are important players in hematologic malignancies. Clinical investigations focused on drugs reversing altered epigenetic events, namely, epi-drugs. Two different types of DNMT inhibitors (DNMTi) represent analogs of nucleoside and non-nucleoside agents. Hypomethylating agents (HMAs) derived from cytidine, namely azacitidine and decitabine, were approved by the FDA for AML and MDS treatment [137]. Guadecitabine, a dinucleotide of decitabine and deoxyguanosine, belongs to the second generation of HMAs. Due to its better incorporation into DNA, guadecitabine exhibits a higher stabilization against cytidine deamination compared to azacitidine and decitabine.

In addition, the novel molecule GSK368503 was identified for the potential reversible and selective inactivation of DNMT1 in preclinical models. The GSK3685032 molecule showed an increased anti-proliferative effect and presented less cytotoxicity compared to decitabine (80% vs. 29% for the negative growth death index) in 51 cell lines derived from leukemia, lymphoma, and multiple myeloma. The application of GSK3685032 resulted in robust DNA hypomethylation and activation of transcription, leading to better survival in mouse models [138].

**Figure 3:**
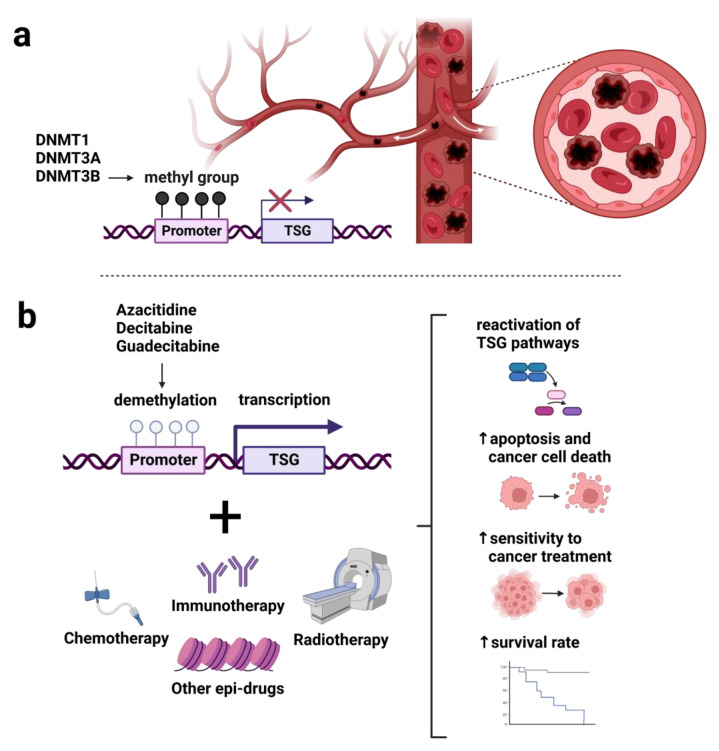
Clinical significance of combined treatment with hypomethylating agents and other therapies in hematologic cancer patients. DNA methyl transferases DNMT1, DNMT3A, and DNMT3B mediated DNA methylation resulting in aberrant gene silencing. In hematologic cancers, aberrant DNA methylation cause changes in the function of tumor suppressor genes, which play a role in cancer-associated pathways (**a**). Epi-drugs, including first-generation azacitidine, decitabine, and second-generation guadecitabine, are used as DNA methylation inhibitors in leukemias, myelodysplastic syndromes, and lymphomas. HMAs aim to reactivate silenced genes subsequently involved in apoptotic, repairing, and angiogenic pathways. Recently, a strategy of combined treatment with HMAs and dose-specific chemotherapy/immunotherapy/radiotherapy and other epi-drugs represents unique challenges in patient outcomes (**b**). Abbreviations: DNMT1, DNA methyl transferase 1; DNMT3A, DNA methyl transferase 3A; DNMT3B, DNA methyl transferase 3B; HMAs, hypomethylating agents; TSG, tumor suppressor gene.

Mounting evidence documented that a combination of HMAs with chemotherapy, immunotherapy, or other treatment modalities can improve targeting, drug processing, and cytotoxicity (Figure 3).

### 4.1. HMAs Monotherapy

In the DACO-016 randomized phase III study for decitabine monotherapy, the median OS was 7.7 months in patients with AML treated with decitabine compared to the control arm with OS of 5.0 months (hazard ratio (HR), 0.85; 95% confidence interval (CI), 0.69–1.04; *p* = 0.1079). The survival rate was similar after an additional year, with a median OS in decitabine-intervened patients compared to controls at 7.7 and 5.0 months, respectively (HR, 0.82; 95% CI, 0.68–0.99; *p* = 0.0373). Furthermore, PFS and response rates were more favorable for decitabine than for other treatments [139]. Accordingly, in a phase III randomized study with azacitidine monotherapy, a longer OS was observed for azacitidine-treated patients compared to standard treatment (10.4 vs. 6.5 months) (HR 0.85; 95% CI, 0.69–1.03; *p* = 0.1009) [140].

In addition to standard subcutaneous and intravenous application of decitabine and azacitidine, oral azacitidine (CC-486) intervention was approved as maintenance therapy. In phase III of the QUAZAR AML-001 study, CC-486 administration to older AML patients previously treated with chemotherapy presented significantly longer median OS (24.7 vs. 14.8 months, *p* < 0.001) and relapse-free survival (10.2 vs. 4.8 months, *p* < 0.001) compared to the placebo group. In addition, adverse effects associated with CC-486 treatment rarely required discontinuation and were successfully managed with a change of dose or using a medication, suggesting a safety profile of CC-486 therapy for AML [141,142]. Due to rapid deamination, oral decitabine can be applicated only in combination with cedazuridine (cytidine deaminase inhibitor). In the phase I study comprising MDS and CMML patients, this combination showed a similar clinical response, safety profile, and demethylation to intravenous decitabine [143].

In phase II of the dose-expansion study, patients with AML received 60 or 90 mg/m^2^ of guadecitabine via subcutaneous administration in the five-day regimen or 60 mg/m^2^ in a ten-day regimen. As noted, higher doses did not show a benefit in terms of clinical outcomes for patients. Although complete response rates were higher in the ten-day regimen compared to the five-day regimen (18.9% vs. 8%; *p* = 0.15), the differences were not statistically significant. Adverse events during treatment occurred mainly during the ten-day regimen [144]. The response rates to guadecitabine in phase I and II studies were 17% and 23%, respectively [145]. In the guadecitabine study on MDS patients, the number of patients who achieved an overall response was similar between dose-differentiated groups (60 vs. 90 mg/m^2^). Moreover, 43% of relapsed or refractory MDS patients previously treated with HMAs achieved an overall response [146].

An overview of ongoing clinical trials focusing on the role of DNA methylation in leukemias and MDS, containing the study of decitabine/cedazuridine treatment with BMS-986253 in MDS patients, is shown in Table 1.

### 4.2. Combined HMAs Therapy with Other Treatments

Azacitidine pretreatment followed by high-dose chemotherapy with cytarabine and mitoxantrone showed an improved response rate in 61% of older AML patients with poor prognoses [147]. Furthermore, a combination of decitabine with vorinostat and chemotherapy led to a 54% overall response rate in pediatric patients with relapsed and refractory AML. The absence of MRD observed in 90% of patients showing 2-year OS for patients without or with adverse effects was 75.6% and 17.9% (*p* < 0.001), respectively [148]. Treatment with azacitidine and venetoclax (Bcl-2 inhibitor) represents a promising therapeutic approach since this combination showed significantly better survival and response rates compared to placebo or intensive chemotherapy-treated AML patients [149,150].

An active anti-cancer response by specific T-lymphocytes was observed in MDS patients who received an intervention with decitabine and the NY-ESO-1 vaccine [151]. However, Holmberg-Thydén et al. did not confirm an improved immune response when combining this vaccine and azacitidine application in high-risk MDS patients, and all patients progressed to AML [152]. Contradictory results were observed in clinical trials that studied a combination of HAMs with immune checkpoint inhibitors. While Saxena et al. and Daver et al. observed an anti-tumor immune response and better survival in AML [153,154], other studies did not find better clinical outcomes for MDS and AML patients compared to HMA treatment alone [155,156].

Nieto et al. studied a combination of azacitidine with high-dose chemotherapy (gemcitabine with busulfan and melphalan) and stem-cell transplantation in refractory/relapsed lymphomas, presenting that event-free survival and OS rates for HL and DLBCL patients were 76% vs. 95% and 65% vs. 77%, respectively [157]. Similar results were observed in a clinical trial with a combination of decitabine and a modified DHAP regimen (cisplatin, cytarabine, dexamethasone) in relapsed DLBCL patients, leading to improved responses and better prognosis [158]. In clinical trial phase II testing, CAR-T immunotherapy in relapsed DLBCL patients pretreated with decitabine, fludarabine, and cyclophosphamide showed a median follow-up of 10.9 months and overall response and CR at 90.9% and 63.6%, respectively. These findings suggested that the application of CAR-T therapy with decitabine pretreatment could be a potentially safe and efficient intervention for DLBCL patients [159].

Ongoing clinical trials involving HMAs in combined therapy aim to determine the optimal therapy dose and synergy effect in the treatment of lymphoma patients (Table 2).

## 5. Conclusions and Future Directions

Current clinical oncology and cancer research aims to find new biomarkers for precise diagnosis and new targets for more efficient anti-cancer treatment modalities. Due to the aggressive nature of hematologic malignancies, early and correct diagnosis is the key to improving prognosis, treatment results, and patient survival. The highly toxic treatment negatively affects the entire organism and develops severe side effects.

Enormous improvements in high-throughput sequencing methods and bioinformatic tools have helped to elucidate the impact of genetics and epigenetics on carcinogenesis. In addition to known genetic alterations, DNA methylation is one of the main epigenetic mechanisms that play a role in cancer initiation and progression. In the future, DNA methylation-based stratification of hematologic patients might lead to more personalized treatment with better outcomes. The reversibility of changes in DNA methylation landscapes enables broad clinical implications. However, adverse events associated with indiscriminate global hypomethylation with DNA methylation inhibitors are a matter of concern, and further investigations are highly warranted. According to the findings, DNA methylation processes in hematologic malignancies are usually associated with other mechanisms of epigenetic regulations, including histone modifications and miRNA regulation. Thus, evaluating the role of epigenetic modifications in a more complex matter would be beneficial for blood cancer patients.

Mounting evidence from clinical trials highlights the potential of therapeutic strategies focusing on reversing aberrant DNA methylation patterns in hematological malignancies. Importantly, a combination of epi-drugs with chemo- or immunotherapy remains an emerging research area. Recent findings suggest that the DNA methylation-based approach sensitizes other anti-cancer treatment modalities and may improve the clinical outcomes for hematologic cancer patients.

## Figures and Tables

**Table 1:** The effect of hypomethylating agents on outcomes of patients with leukemias and myelodysplastic syndromes. The table summarizes the list of active and recruiting clinical trials focused on adverse events, treatment efficacy, EFS, PFS, OS, and ORR (according to https://clinicaltrials.gov/ and https://beta.clinicaltrials.gov/, accessed on 1 December 2022).

Study	Design	Disease	Purpose	Patients	Intervention	Status
NCT02935361	An interventional open-label study single group assignment	CMML MDS AML	To assess the potential risk of treatment-related adverse events and to evaluate ORR and OS	33 adults	Guadecitabine and atezolizumab as a humanized monoclonal antibody every 28 days up to 2 years in the absence of disease toxicity	Active, not recruiting
NCT04167917	An interventional open-label study single group assignment	AML MDS CML	To confirm the tolerability and safety of the oral HMAs and to evaluate the CBR, OS, PFS, and ORR after epi-drug therapy	20 adults	DNMT1 inhibitor known as NTX-301 with previously shown anti-leukemic efficacy	Recruiting
NCT05042531	An interventional prospective randomized study parallel assignment	AML	To determine the incidence of adverse events developed during the first and second cycles of azacitidine treatment	30 adults	Low-dose dasatinib with azacitidine as a maintenance therapy option in chemotherapy-treated patients	Recruiting
NCT02878785	An interventional randomized open-label study factorial assignment	AML	To test the best dose and assess the treatment-related adverse events	25 adults	A combination of decitabine (5 days every month) with talazoparib (daily oral administration)	Active, not recruiting
NCT03164057	An interventional randomized open-label study parallel assignment	AML MDS	To analyze OS, EFS, and non-hematologic toxic events in patients receiving DNMTi before the chemotherapy	206 adults/ children	Intravenously administered azacitidine or decitabine before chemotherapy regimens	Active, not recruiting
NCT04722952	An interventional open-label study single-group assignment	AML	To define the efficacy of combined therapies in refractory/relapsed patients and evaluate CR, ORR, OS, EFS, and PFS	30 adults	PD-1 monoclonal antibody visilizumab plus azacitidine, and a homoharringtonine regimen	Recruiting
NCT01806116	An interventional open-label study single group assignment	MDS AML	To investigate the impact on posttransplant outcomes and evaluate the incidence of GvHD	30 adults/ children	Intravenous administration of decitabine followed by allogeneic HSCT	Unknown
NCT02684162	An interventional open-label study single-group assignment	AML CML MDS	To confirm CRR, RFS, safety, and toxicity of treatment with/without donor lymphocyte infusion	55 adults	Guadecitabine and donor lymphocyte infusion in case of the absence of disease progression or toxicity	Active, not recruiting
NCT03080766	An interventional open-label study single-group assignment	AML	To identify CR, DFS, and OS in patients with chromosomal abnormalities associated with bad prognosis	20 adults	Decitabine treatment with the examination of bone marrow after each therapy course Afterward, patients will receive HSCT plus decitabine.	Recruiting
NCT05426798	An interventional open-label study single-group assignment	AML MDS	To test the tolerability and anti-tumor efficacy of combined treatment in patients with recurrent/refractory disease	73 adults	Injection of monoclonal antibody TQB2618 with azacytidine/decitabine	Recruiting
NCT03298321	An observational prospective study	AML	To study the potential effect of epi-drug on arrhythmia and left atrium fibrosis	14 adults	Azacitidine administration	Unknown
NCT03066648	An interventional non-randomized study parallel assignment	AML	To characterize doses for monoclonal antibodies in combination with HMAs and to evaluate dose-limiting toxicities, ORR, PFS, and TTP	243 adults	Decitabine in combination with MBG453 and/or PDR001 as monoclonal antibodies, or azacitidine together with MBG453	Active, not recruiting
NCT03238248	An interventional open-label study single-group assignment	MDS	To reveal the efficacy of combined treatment in patients who failed primary therapy with DNMTi and to determine CR and PFS	71 adults	Azacitidine infusion and antineoplastic pevonedistat as an inhibitor of the Nedd8 activating enzyme	Active, not recruiting
NCT02131597	An interventional open-label study single-group assignment	MDS	To determine CRR, OS, EFS adverse events, and mortality rate in study participants	107 adults	Administration of guadecitabine up to 24 courses in the absence of progression and toxicity	Active, not recruiting
NCT05148234	An interventional non-randomized open-label study sequential assignment	MDS	To evaluate the efficacy, safety, and tolerability of treatment and determine ORR, CRR, and therapy-associated adverse events	200 adults	BMS-986253 plus decitabine/cedazuridine	Recruiting
NCT04187703	An interventional open-label study single group assignment	MDS	To analyze ORR, CRR, improvement, and specify treatment-associated adverse events	20 adults	Low doses of azacitidine and decitabine	Recruiting

Abbreviations: AML, acute myeloid leukemia; CBR, clinical benefit rate; CML, chronic myelomonocytic leukemia; CMML, chronic myelomonocytic leukemia; CR, complete remission; CRR, complete response rate; DFS, disease-free survival; DNMT, DNA methyltransferase; DNMTi, DNA methyltransferase inhibitor; EFS, event-free survival; GvHD, graft-versus-host disease; HMAs, hypomethylating agents; HSCT, hematopoietic stem cell transplantation; MDS, myelodysplastic syndromes; OS, overall survival; ORR, objective response rate; PD-1, programmed cell death-1; PFS, progression-free survival, RFS, relapse-free survival; TTP, time of progression.

**Table 2:** The impact of combined therapy with hypomethylating agents and other treatments on the outcomes of lymphoma patients. The table summarizes the list of active and recruiting clinical trials focused on adverse events, treatment efficacy, and survival rates (according to https://clinicaltrials.gov/ and https://beta.clinicaltrials.gov/, accessed on 1 December 2022).

Study	Study Design	Disease	Purpose	Patients	Intervention	Study Status
NCT04510610	An interventional open-label study single-group assignment	HL	To define the response duration and PFS in patients with relapsed/refractory disease treated with camrelizumab plus decitabine	100 adults/ children	Decitabine plus anti-PD-1 monoclonal antibody camrelizumab	Recruiting
NCT04233294	An interventional open-label study single-group assignment	HL	To document the adverse effects of combined therapy and ORR in cancer patients	100 adults/ children	HDAC inhibitor chidamide in combination with monoclonal antibody camrelizumab plus decitabine	Recruiting
NCT03250962	An interventional randomized open-label study parallel assignment	HL	To test the safety of decitabine with SHR-1210 therapy compared to SHR-1210 monotherapy and investigate treatment-related adverse events	280 adults/ children	SHR-1210 as an anti-PD-1 monoclonal antibody with/without the decitabine in relapsed or refractory patients	Recruiting
NCT04337606	An interventional randomized open-label study	NHL	To assess the safety, treatment efficacy, and therapy-induced adverse events	100 adults	Decitabine plus HDAC inhibitor chidamide or monoclonal antibody camrelizumab.	Recruiting
NCT04514081	An interventional randomized open-label study parallel assignment	HL	To compare ORR and adverse events between immunotherapy-resistant patients with chidamide+decitabine+camrelizumab treatment vs. decitabine+camrelizumab	200 adults/ children	Decitabine with monoclonal antibody camrelizumab or as a combination with camrelizumab+HDAC inhibitor chidamide.	Recruiting
NCT03494296	An observational case-control prospective study	DLBCL	To determine the efficacy and safety of low-dose decitabine with a chemotherapy regiment in relapsed and refractory patients	150 adults	A combination of chemotherapeutics cyclophosphamide+vindesine+bonisone with decitabine	Recruiting
NCT03346642	An interventional open-label study single-group assignment	DLBCL	To document potential treatment-related adverse events, ORR, CR, PFS, and OS in cancer patients	30 adults	Combined therapies with gemcitabine, vinorelbine, doxorubicin, and monoclonal antibody SHR-1210 with/without decitabine	Unknown
NCT05320640	An interventional open-label study single-group assignment	NHL solid tumors	To evaluate predictive biomarkers of response, adverse events, ORR, the safety, and efficacy of combined therapy in participants with relapsed/refractory NHL and advanced solid tumors	100 adults/ children	Chemotherapy-free regimen with HDAC inhibitor chidamide, low-dose hypomethylating agent decitabine, and anti-PD-1/PD-L1/CTLA-4 antibodies	Recruiting
NCT03445858	An interventional open-label study single-group assignment	Lymphoma solid tumors	To detect feasibility, toxicities, tolerability, and anti-tumor effect of combined therapy	21 adults/ children	Pembrolizumab with decitabine and fixed-dose hypofractionated radiotherapy	Active, not recruiting
NCT04553393	An interventional randomized open-label study parallel assignment	NHL	To assess the effect of combined therapies in relapsed/refractory participants and reveal adverse events, PFS, and OS after the intervention	80 adults/ children	A combination of decitabine-primed Tandem CAR19/20 engineered T cells with/without chidamide, decitabine	Recruiting

Abbreviations: anti-PD-1, anti-programmed cell death-1; CR, complete response; CTLA-4, cytotoxic T-lymphocyte–associated antigen 4; DLBCL, Diffuse large B-cell lymphoma; HDAC, histone deacetylase; HL, Hodgkin lymphoma; NHL, non-Hodgkin lymphoma; OS, overall survival; PD-L1, programmed death-ligand 1; ORR, objective response rate; PFS, progression-free survival.

## Data Availability

Not applicable.
